# Association of urinary prostaglandin E2 metabolite and mortality among adults

**DOI:** 10.1038/s41598-022-23773-x

**Published:** 2022-11-07

**Authors:** Wanqing Wen, Gong Yang, Qiuyin Cai, Xiao-Ou Shu, Wei Zheng

**Affiliations:** Division of Epidemiology, Department of Medicine, Vanderbilt University Medical Center, Nashville, TN USA

**Keywords:** Diseases, Medical research, Risk factors

## Abstract

Prostaglandins play a critical role in inflammatory response. To investigate the association of urinary PGE-M, a stable end-product of prostaglandin E2 (PGE_2_) with overall and cause-specific mortality and examine potential effect modifiers, we obtained urinary PGE-M levels of 2927 non-cancerous adults from our previous case-control studies nested in the Shanghai Women’s Health Study and Shanghai Men’s Health Study, two cohort studies conducted in Shanghai, China. Mortality data and modifiable factors associated with urinary PGE-M were obtained from the parent cohort studies. Using linear regression models, we found that high urinary PGE-M levels were significantly associated with low education, heaving smoking, old age at urine collection, and abdominal obesity. Using Cox proportional hazards models, we found that increase (per standard deviation) of urinary PGE-M levels were significantly associated with overall mortality (adjusted hazard ratio = 1.19, 95% confidence interval: 1.07, 1.33) and particularly deaths from cardiometabolic diseases (adjusted hazard ratio = 1.27, 95% confidence interval: 1.11, 1.44). The increased death risks persisted across different time intervals during the follow-up and were stronger among participants who were younger than 60 (*P* = 0.0014 for all- cause mortality and *P* = 0.007 for deaths from cardiometabolic diseases) at urine collection or perhaps among those who had higher education.

## Introduction

Prostaglandins (PGs) play a critical role in the generation of the inflammatory response^1^. PGE_2_ is one of the most abundant PGs produced in the body and is involved in all processes leading to the classic signs of inflammation. This metabolite is an important mediator of many biological functions, such as regulation of immune responses, blood pressure, gastrointestinal integrity, and fertility.

PGE_2_ is an unstable compound that is rapidly metabolized in vivo to a stable metabolite, 11 a-hydroxy-9,15-dioxo-2,3,4,5-tetranorprostane-1,20-dioic acid (PGE-M). Thus, measurement of excreted urinary PGE-M has been used to quantify systemic PGE_2_ production in vivo. Urinary level of PGE-M is considered to have a more complete capture of prostaglandin production, as it will reflect a combination of prostaglandins from both the blood stream and the kidney^2,3^. We have previously reported the association between urinary PGE-M levels and multiple cancer risks.

In addition to cancers, it has become widely accepted that chronic inflammation is a driving force behind many other chronic diseases^1,4–6^, including ischemic heart disease, stroke, and diabetes mellitus, which are the major causes of death in the world today^7^. Over the past decade, we have conducted multiple nested case-control studies^8–13^ to investigate the association between urinary PGE-M levels and multiple cancer risks and found the individuals with higher urinary PGE-M levels had increased risks of colorectal cancer^8^, gastric cancer^12^, breast cancer^10^, and pancreatic cancer^11^.

In addition, the detrimental effects of chronic inflammation on health are now known to persist throughout the life span to increase adulthood mortality. Characterizing the association of chronic inflammation with health risk and life span should thus also be a top public health priority. To this end, we investigated the modifiable factors associated with urinary PGE-M levels and test whether the latter were associated with premature death in this study.

## Methods

### Study population

The 2927 non-cancerous adults included in this study consisted of controls from our previously case-control studies nested in the Shanghai Women’s Health Study (SWHS) and Shanghai Men’s Health Study (SMHS), two ongoing prospective cohort studies conducted in Shanghai, China. Mortality data and modifiable factors associated with urinary PGE-M were obtained from the parent cohort studies. The parent studies were approved by the institutional review boards of all participating institutions. We confirm that all methods were carried out in accordance with relevant guidelines and regulations. Detailed descriptions of study design and methods have been published elsewhere^14,15^. Briefly, participants were recruited from typical urban communities in Shanghai, China. The SWHS recruited 74,941 women aged 40–70 years from 1996 to 2000 with a 92.7% participation rate in the baseline survey. The SMHS recruited 61,480 men aged 40–74 years from 2002 to 2006 with a 74.0% participation rate in the baseline survey. At the time of enrollment, each participant signed consent and completed an in-person survey conducted by trained interviewers. Of the study participants, 65,754 (88%) women and 54,769 (89%) men provided a spot urine sample. Urine samples were collected into a sterilized cup containing 125 mg ascorbic acid to prevent oxidation of labile metabolites. After collection, the samples were kept in a portable Styrofoam box with ice packs (at approximately 0–4 °C) and processed within 6 h for long term storage at − 70 °C. All participants also filled out a biospecimen collection form at the time of sample collection, which included the date and time of sample collection, time of last meal, and use of any medications during the previous week.

Controls of our previous reported nested case-control studies were randomly selected from cohort members and individually matched to each case by age at sample collection (within 2 years), sex, time of sample collection (morning or afternoon), date of sample collection (within 1 month), and time interval since last meal (within 2 h). Controls were free of cancer at the time of cancer diagnosis of their corresponding case. The total sample from the previous nested case-control studies consisted of 5726 adults, including 2799 cancer cases (537 lung, 275 pancreatic, 368 stomach, 603 colorectal, 597 breast, 120 ovarian, 160 corpus uteri, and 88 other cancers) and 2927 non-cancer controls. We excluded all cancer cases from this study. Among the remaining 2927 non-cancer individuals, 2276 women and 651 men were participants from the SWHS and the SMHS respectively.

### Urinary PGE-M measurement

Urinary PGE-M (11-α-hydroxy-9,15-dioxo-2,3,4,5-tetranor-prostane-1,20-dioic acid) level was measured using a liquid chromatography/tandem mass spectrometric method as described in previous studies^2,8–13^. Briefly, 0.75 mL urine was acidified to pH 3 with HCl and endogenous PGE-M was then converted to the *O*-methyloxime derivative by treatment with methyloxime HCl. The methoximated PGE-M was extracted, applied to a C-18 Sep-Pak, and eluted with ethyl acetate. Liquid chromatography was conducted on a Zorbax Eclipse XDB-C18 column attached to a ThermoFinnigan Surveyor MS Pump (Thermo Finnigan). For endogenous PGE-M, the predominant product ion *m*/*z* 336 representing [M-(OCH_3_ + H_2_O)]^−^ and the analogous ion, *m*/*z* 339 [(M-OC[^2^H_3_] + H_2_O)]^−^ for the deuterated internal standard, was monitored in the selected reaction monitoring (SRM) mode. Quantification of endogenous PGE-M used the ratio of the mass chromatogram peak areas of the m/z 336 and *m*/*z* 339 ions. The lower limit of detection of PGE-M was in the range of 40 pg, approximately 100-fold below levels in normal human urine. The coefficients of variation varied from 2.8 to 10.8% across the studies for samples analyzed within batches. Urinary creatinine levels were measured using a test kit from Sigma Company. The PGE-M levels were reported as ng PGE-M/mg creatinine. L﻿aboratory staff was blinded to the case-control status of urine samples and the identity of quality control samples included in the studies.

### Outcome ascertainment

Death information was obtained through the follow-up surveys and by linkage to the database of the Shanghai Death Registry. The underlying of each death was assigned according to the international classification of diseases (9th version). The end date of the observation in this analysis was set as the date of death for deceased cohort members or the date of the last follow-up or June 30, 2016 for those who were still alive, half a year ahead of the last annual linkage with the Shanghai Death Registry, whichever was earlier. Cause-specific mortality categories were grouped according to the ICD-9 codes and were classified as diabetes (ICD-9 250), diseases of circulatory system (ICD-9 401-459), and all other causes.

### Statistical analyses

The distribution of urinary creatinine-adjusted PGE-M levels was skewed to the high values and thus log-transformation was used to improve the normality. We used linear regression models to evaluate changes of log-transformed PGE-M levels by selected demographic or lifestyle factors and medication use during the previous week of sample collection. Thus, the exponential of regression coefficients represents the ratio of geometric means for categorical variables as compared with the reference group or for each unit increment of a continuous variable. The selected covariates were included in the same linear models for mutual adjustment.

The hazard ratios (HR) and 95% confidence intervals (CI) for the association of urinary PGE-M levels and subsequent overall or cause-specific mortalities were analyzed using Cox proportional hazards models stratified by batches of urinary PGE-M assay. We used the age as the time scale with left truncation at age of sample collection. The proportional hazards assumption was evaluated with the Schoenfeld residuals. We used the restricted cubic spline function for urinary PGE-M levels to examine the linearity of its association with the mortalities^16^. The cause-specific mortality was analyzed in the competing risks context using the cause-specific hazards functions approach^17^. Potential confounders, including age at sample collection, education levels, income, use of NSAID or antibiotics one week before urinary sample collection, alcohol drinking status (ever/never), smoking pack-years, BMI and WHR, were included in the regression models for adjustment.

### Ethics approval and consent to participate

The protocol was approved by the Committee of Ethical Research from the Vanderbilt University, and written informed consent was obtained from all participants.

## Results

Among the 2927 participants included in this analysis, the median (range) age at urine sample collection was 59.7 (49.7–66.3) years and the median (range) of follow-up periods was 17.4 (14.3–18.6) years. A total of 361 (12.3%) participants died during the follow-up [36 (1.2%) of diabetes, 211 (7.2%) of diseases of circulatory system, and 114 (3.9%) of other causes]. Both diabetes and cardiovascular diseases are metabolic disorders and were thus were grouped together as cardiometabolic diseases in the subsequent analysis of cause-specific mortality.

Table 1 presents the frequencies of selected demographic and lifestyle factors by gender and the association of these factors with log-transformed PGE-M levels. Of note, individuals with education less than middle school, without use of NSAID during the week preceded the urine collection, with smoking pack-years > 40 had significantly increased PGE-M levels as compared with the respective reference groups, so did the individuals with older age and higher waist-hip ratio.

**Table 1:** Frequency distributions of selected participants’ characteristics and their associations with urinary PGE-M levels.

	Women (N = 2276)	Men (N = 651)	All participants (N = 2927)		
	%	%	%	Ratio (95% CI)*	p
**Education**					
More than high school	12.2	26.6	15.4	Reference	
High school	22.2	28.1	23.5	1.00 (0.93, 1.07)	0.955
Middle school	32.5	31.0	32.1	0.98 (0.92, 1.05)	0.649
Less than middle school	33.1	14.3	28.9	1.08 (1.00, 1.17)	0.044
**Income**					
Low	19.6	8.6	17.2	Reference	
Median	37.1	47.9	39.5	0.98 (0.92, 1.04)	0.561
High	43.3	43.4	43.4	0.94 (0.88, 1.01)	0.069
**Regular physical activity**					
No	58.3	46.5	55.7	Reference	
Yes	41.7	53.5	44.3	0.99 (0.94, 1.03)	0.524
**Smoking pack-years**^**†**^					
0	97.0	43.0	85.0	Reference	
< = 20	3.0	22.9	7.4	1.01 (0.93, 1.11)	0.789
< = 40		22.7	5.1	1.05 (0.94, 1.18)	0.383
> 40		11.4	2.5	1.22 (1.05, 1.42)	0.008
**Ever had alcohol-drinking**					
No	97.5	66.4	90.6	Reference	
Yes	2.5	33.6	9.4	1.04 (0.96, 1.13)	0.320
**NSAID use**^**‡**^					
No	90.3	86.2	89.4	Reference	
Yes	9.7	13.8	10.6	0.81 (0.76, 0.87)	6.5 × 10^–9^
**Antibiotics use**^**‡**^					
No	93.6	95.1	93.9	Reference	
Yes	6.4	4.9	6.1	0.96 (0.88, 1.05)	0.378

	Median(IQR)	Median(IQR)	Median(IQR)		
Age at sample collection	57.7 (48.6, 64.9)	66.0 (55.9, 71.1)	59.7 (49.7, 66.3)	1.04 (1.03, 1.06)	6.8 × 10^–9^
Body mass index	24.1 (22.0, 26.6)	23.8 (21.8, 25.6)	24.0 (21.9, 26.5)	1.01 (0.99, 1.04)	0.237
Waist-hip ratio	0.82 (0.78, 0.85)	0.90 (0.86,0.94)	0.83 (0.79, 0.88)	1.06 (1.03, 1.09)	5.4 × 10^–5^

IQR interquartile range.

*Ratio of geometric means with mutual adjustment for all variables listed in the table. It was estimated as compared with the reference group for categorical variables, or for each 5-year increment for age at sample collection, or for each standard deviation increment for BMI and WHR.

^†^For women, smoking pack-years were defined as 0 or > 0.

^‡^Ever used NSAID or antibiotics one week before urinary sample collection.

**Figure 1:**
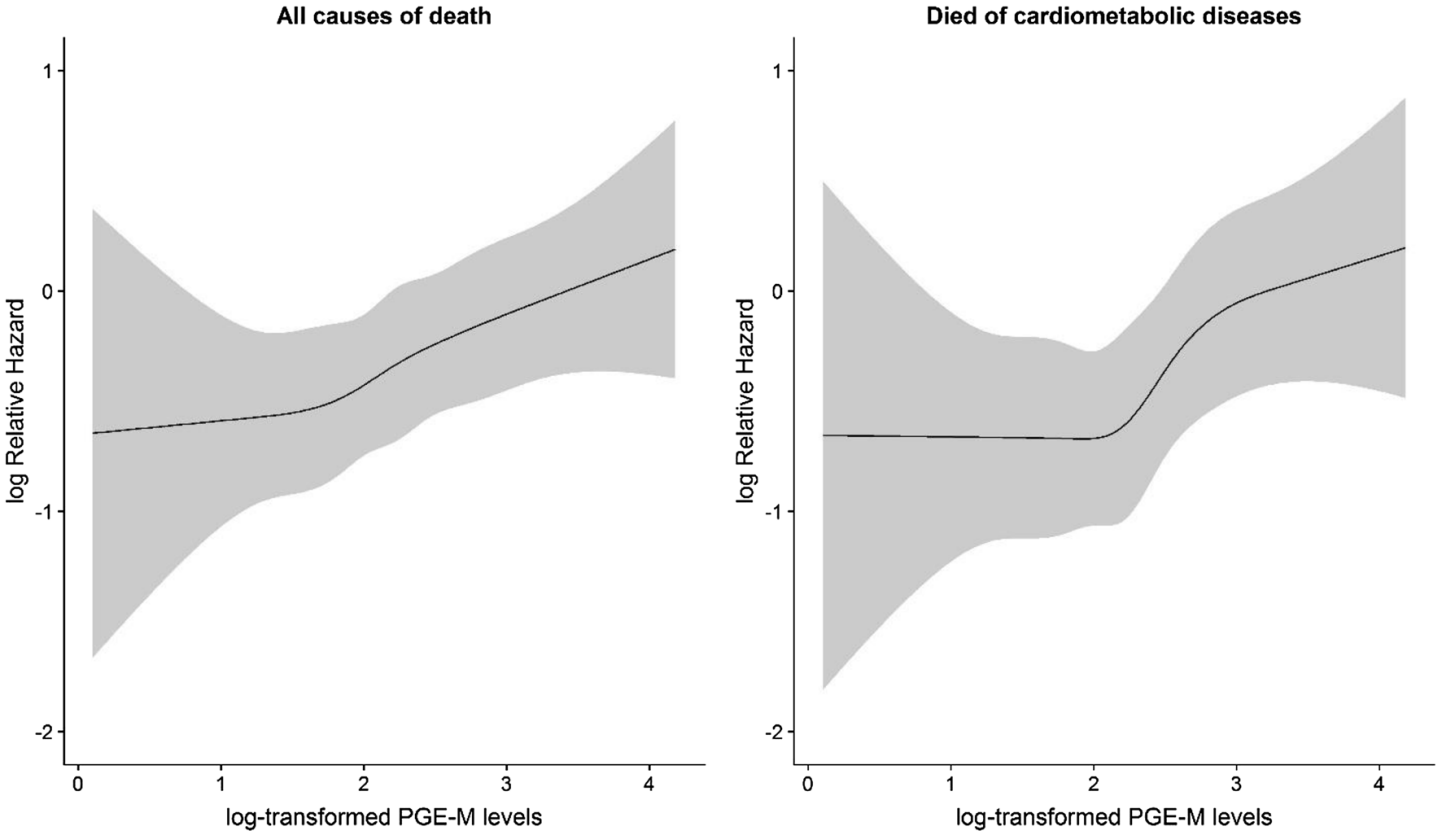
Associations of urinary PGE-M levels with all-cause mortality (left) and death from cardiometabolic diseases (right) using the restrict cubic spline function to account for non-linearity.

A significant linear association of urinary PGE-M levels with risk of all-cause mortality and deaths from cardiometabolic diseases was shown in Fig. 1. The statistical tests for non-linearity of the association based on the restricted cubic spline function were not significant with *P* = 0.9418 for all-cause mortality and *P* = 0.3737 for deaths from cardiometabolic diseases. Table 2 presents the adjusted association of death with categorized and continuous (for each standard deviation increment) PGE-M levels. As compared with the first quintile of PGE-M, higher levels of urinary PGE-M were associated with higher risk of deaths with highly significant linear trend (HR = 1.19, 95%CI 1.07, 1.33. *P* = 0.001 for all-cause mortality and HR = 1.27, 95%CI 1.11, 1.44, *P* = 0.0003 for death from cardiometabolic diseases). Urinary PGE-M levels were not significantly associated with deaths from the other causes.

**Table 2:** Association between urinary PGE-M levels and death.

PGE-M levels	All-cause mortality			Died of cardiometabolic diseases			All other causes of death		
	No. of deaths	HR (95% CI)*	P	No. of deaths	HR (95% CI)*	P	No. of deaths	HR (95% CI)*	P
Quintile 1	62	Reference		42	Reference		20	Reference	
Quintile 2	67	1.32 (0.93, 1.88)	0.125	43	1.25 (0.81, 1.93)	0.323	24	1.45 (0.79, 2.68)	0.231
Quintile 3	74	1.36 (0.96, 1.93)	0.083	45	1.23 (0.80, 1.91)	0.342	29	1.60 (0.89, 2.88)	0.118
Quintile 4	62	1.19 (0.83, 1.70)	0.351	42	1.22 (0.79, 1.89)	0.364	20	1.11 (0.59, 2.09)	0.739
Quintile 5	96	1.73 (1.25, 2.40)	0.0009	75	2.01 (1.37, 2.95)	0.0004	21	1.17 (0.63, 2.18)	0.612
Continuous	361	1.19 (1.07, 1.33)	0.001	247	1.27 (1.11, 1.44)	0.0003	114	1.03 (0.85, 1.26)	0.745

*HRs and 95 CIs for continuous urinary PGE-M (log-transformed) were estimated for the increment by one standard deviation, with adjustment for covariates in Table 1. For cause-specific death, HRs and 95% CIs were derived from the cause-specific hazard models to account for competing risks from other causes.

**Figure 2:**
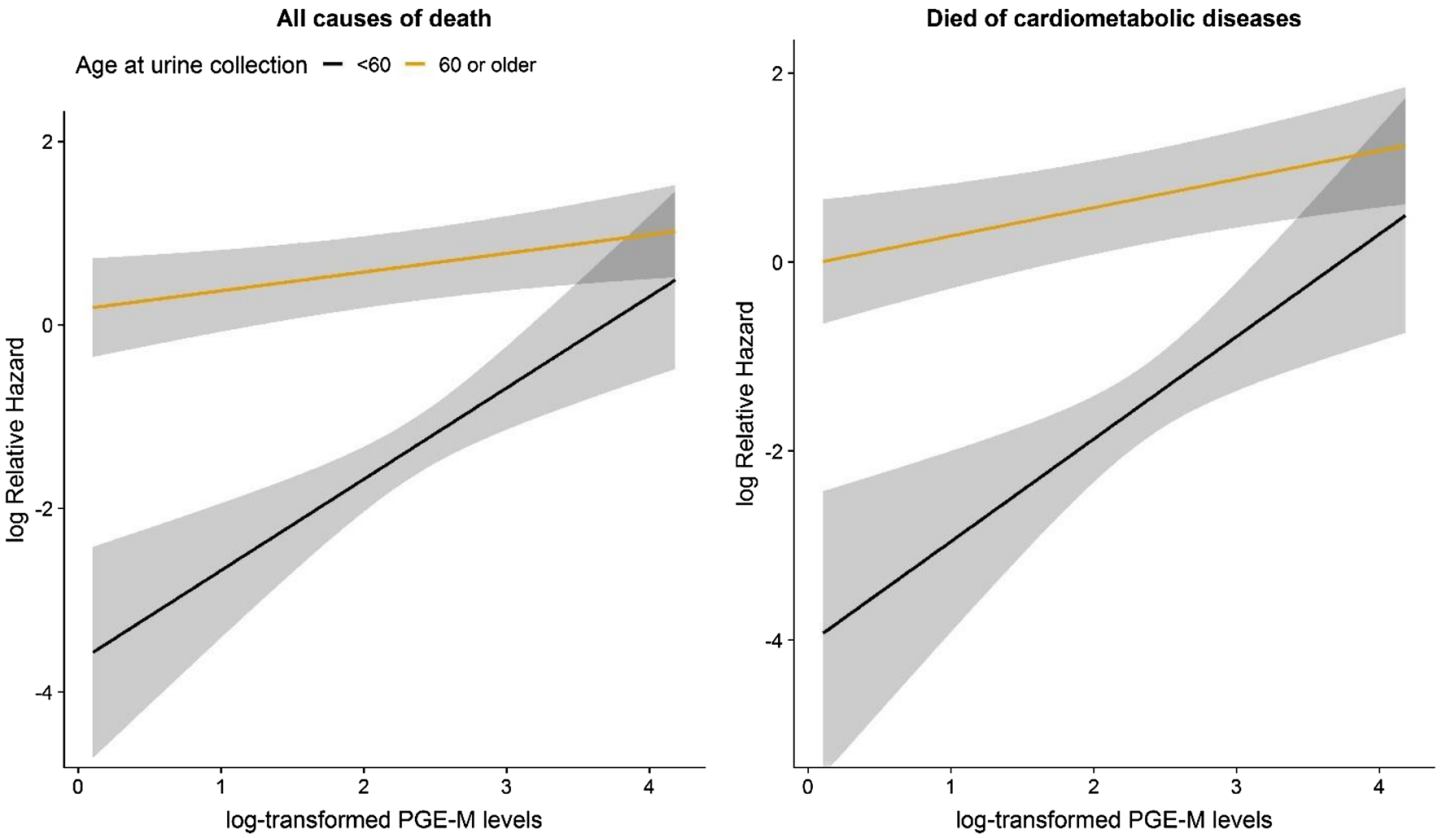
Linear associations of urinary PGE-M levels with all-cause mortality (left) and death from cardiometabolic diseases (right) by age at urine collection (< 60 vs. >  = 60 years).

As shown in Table 3 and Fig. 2, the adjusted associations between urinary PGE-M levels and all-cause mortality were significantly stronger among participants who had younger age (< 60) at urine sample collection than among those who were older (> = 60) with HR = 2.24, 95%CI 1.56, 3.23 vs. HR = 1.13, 95% CI 1.00, 1.26, *P* for heterogeneity test = 0.0014. A similar pattern was observed for the associations for deaths from cardiometabolic diseases with HR = 2.45, 95%CI 1.47, 4.07 vs. HR = 1.21, 95% CI 1.06, 1.38, *P* for heterogeneity test = 0.007. We also observed marginally significantly stronger associations (*P* for heterogeneity test = 0.054 for all-cause mortality and p for heterogeneity test = 0.116 for deaths from cardiometabolic diseases) among participants who had higher education (> = high school) than among those who had less than high school education.

**Table 3:** Association between urinary PGE-M levels and death, stratified by selected covariables.

	All-cause mortality			Died of cardiometabolic diseases		
	No. of deaths	HR (95% CI)*	P	No. of deaths	HR (95% CI)*	P
**Gender**						
Women	253	1.15 (1.01, 1.31)	0.035	173	1.25 (1.06, 1.46)	0.007
Men	108	1.21 (0.98, 1.49)	0.076	74	1.32 (1.07, 1.63)	0.010
P for interaction			0.690			0.679
**Education levels**						
< High school	270	1.10 (0.97, 1.25)	0.137	183	1.18 (1.02, 1.37)	0.026
> = High school	91	1.42 (1.13, 1.79	0.0026	64	1.52 (1.15, 2.00)	0.003
P for interaction			0.054			0.116
**NSAID use**						
No	321	1.21 (1.08, 1.36)	0.00093	221	1.28 (1.12, 1.47)	0.0003
Yes	40	1.16 (0.76, 1.78)	0.4777	26	1.12 (0.74, 1.71)	0.585
P for interaction			0.869			0.557
**Smoking status**						
Never smoked	291	1.20 (1.06, 1.35)	0.0031	201	1.28 (1.11, 1.49)	0.001
Ever smoked	70	1.19 (0.91, 1.56)	0.198	46	1.27 (0.97, 1.68)	0.084
P for interaction			0.908			0.963
**Age at sample collection**						
< 60	35	2.24 (1.56, 3.23)	1.0e-5	20	2.45 (1.47, 4.07)	0.0006
> = 60	326	1.13 (1.00, 1.26)	0.041	227	1.21 (1.06, 1.38)	0.006
P for interaction			0.0014			0.007
**WHR values**						
< = 0.82 (median)	111	1.14 (0.91, 1.42)	0.261	77	1.2 5 (0.97, 1.62)	0.091
> 0.82 (median)	250	1.20 (1.06, 1.36)	0.0048	170	1.27 (1.10, 1.48)	0.001
P for interaction			0.547			0.901
**Follow-up time**						
During the first 5-year	47	1.18 (0.89, 1.57)	0.244	31	1.34 (0.96, 1.87)	0.091
Between 5 and 10 years	98	1.17 (0.97, 1.43)	0.108	61	1.27 (1.00, 1.61)	0.053
Over 10 years	216	1.21 (1.05, 1.39)	0.009	155	1.25 (1.06, 1.48)	0.007
P for interaction			0.971			0.945

*HRs and 95 CIs for continuous urinary PGE-M (log-transformed) were estimated for the increment by one standard deviation, with adjustment for covariates in Table 1.

The associations between urinary PGE-M levels and death were generally consistent across gender, NSAID use, smoking status, levels of waist-hip ratio, and different time intervals during the follow-up (Table 3).

**Figure 3:**
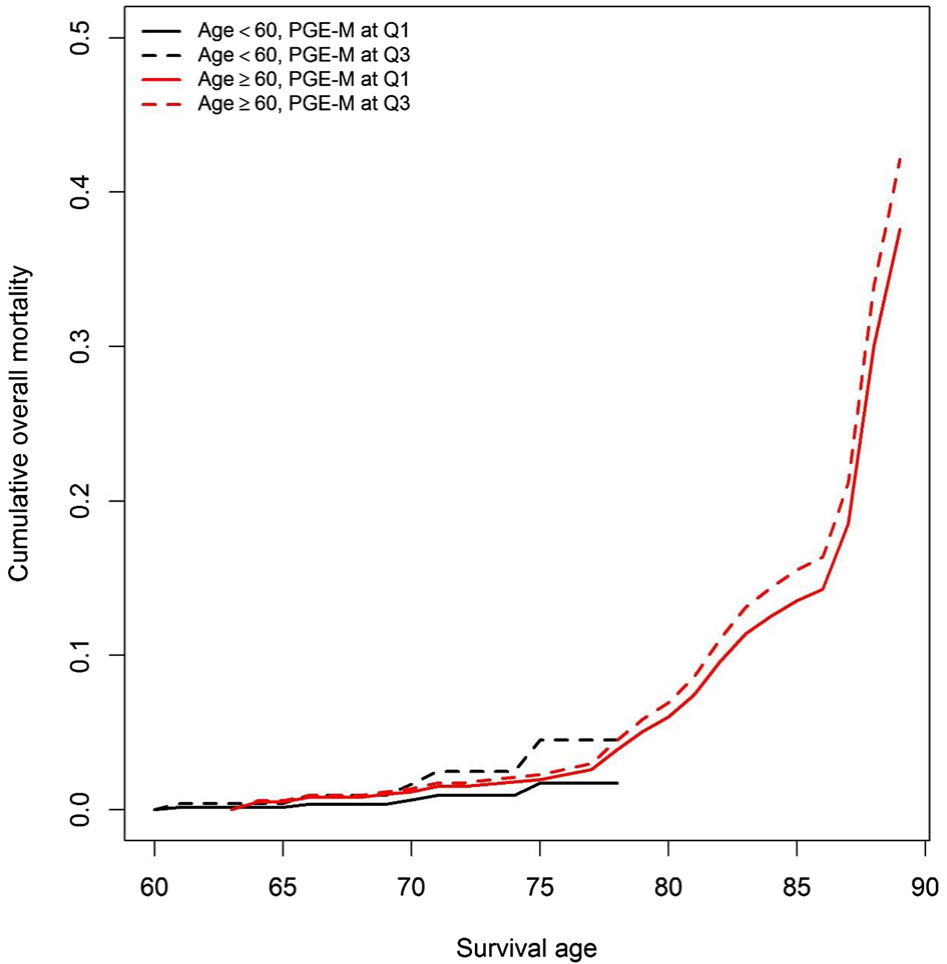
Cumulative mortality associated with urinary PGE-M levels [the first quartile (Q1) vs. the third quartile (Q3)] by age at urine collection (< 60 vs. >  = 60 years).

Figure 3 shows the cumulative overall mortality at 25% (Q1) and 75% (Q3) percentiles of urinary PGE-M levels by age at urine collection (< 60 vs. >  = 60) with adjustment for the covariables. Consistent with the results shown in Table 3 and Fig. 2, higher urinary PGE-M levels were associated with higher cumulative overall mortality, which was more evident among participants who were younger than 60 at urine collection. Similar patterns were observed when plotting curves for the cumulative mortality from cardiometabolic diseases.

## Discussion

In this prospective study, we found that high concentrations of urinary PGE-M were positively related with low education, heaving smoking, old age at urine sample collection, and abdominal obesity as measured with waist-hip ratio. After adjustment for these covariates, we observed that higher concentrations of urinary PGE-M were still associated with a significantly increased all-cause mortality and particularly diabetes and CVD mortality. These positive association was consistent across different time intervals since study enrollment and were stronger among participants who were younger than 60 at urine collection or marginally significantly higher among those who had higher education.

Previous studies reported that low socioeconomic status^18^, smoking^19,20^, older age^21,22^ and obesity^23,24^ were positively associated with chronic inflammation. The chemical constituents of tobacco smoke could induce cyclooxygenase-2 (COX-2) and PGE_2_ synthesis^25^. Aging and obesity are characterized by chronic inflammation. The inflammatory status becomes pervasive over time owing to the exposure to a variety of stressors in older people^21^. Obesity increases the release of pro-inflammatory cytokines and induces multiple components of an inflammatory state^26^. It is well established that biomarkers of inflammation are robust predictors of obesity and morbidity (chronic diseases)/mortality in older people^26–28^. Low socioeconomic status could affect inflammation due to chronic stress, obesity, unhealthy diet, and physical inactivity in people experiencing socioeconomic adversity^18^.

As an inflammatory marker^1^, PGE-M was observed to be positively associated mortality, mainly from cardiometabolic diseases in this study. It is well documented that inflammation plays an important role in the etiology of these diseases^18,29,30^. A recent study revealed that COX-2/PGE_2_ signaling was involved in the regulation of IL-1β auto-stimulation, thus forming an IL-1β/COX-2/PGE_2_ pathway loop, which may result in the high inflammation level and play a pivotal role in the pathogenesis of type 2 diabetes^31^. PGE_2_ plays a key role in the pathogenesis of cardiovascular diseases. It was reported^29^ that a deficiency of PGE_2_ synthase-1 reduced plaque burden in fat-diet low-density lipoprotein receptor knockout mice, and PGE_2_ synthase-1 knockout mice showed impaired left ventricular contraction after acute myocardial infarction. These findings indicate that PGE_2_ plays a pivotal role in cardiovascular inflammation.

We previously reported positive associations of PGE-M with multiple cancers including colorectal cancers^8^, gastric cancer^12^, breast cancer^10^, and pancreatic cancer^11^. Other studies also reported similar findings^32–34^. We did not include cancer patients in the current analysis, as their inclusion might introduce bias in the overall mortality analysis.

We observed consistent PGE-M-death association between early and late periods of the follow-up, which demonstrates the importance of chronic inflammation on premature mortality, emphasizing on controlling for chronic inflammation throughout the life span.

We found that the positive associations were weaker among participants who were older than 60 at urine collection or perhaps among those who had low education. Older people become more vulnerable a wide range of diseases due to declines in biological function and the accretion of cumulative risk factors^35^. Similarly, the increased health burden due to a constellation of biologic, behavioral, and psychosocial risk factors that are more prevalent in people with low socioeconomic status^36^. These factors hence should attenuate the influence of PGE-M on mortality.

Our study’s strengths include its prospective design and adjustment for multiple potential confounders, which minimized the selection bias and confounding. In addition, our study has relatively large sample size and long period of follow-up time. To our knowledge, it comprises the first prospective study to directly evaluate the association of PGE-M with mortality with decent statistical power. Our study provided the direct evidence that PGE-M was associated with increased mortality. There may be a concern that the results from this study were based on a single measurement of a spot urine sample of PGE-M. Ideally, the study hypothesis would be bolstered if multiple measurements of urine samples collected at several different time points were available. Therefore, the association of PGE-M and mortality may have been underestimated due to the less than optimal exposure measurement.

## Conclusion

We found in this study that multiple modifiable factors such as low education, heaving smoking, and abdominal obesity were associated with increased inflammatory responses, as measured with urinary PGE-M concentrations. Higher concentration of urinary PGE-M was associated with increased mortality. We believe that improving lifestyles, e.g., stopping smoking and controlling body weight, would help decrease premature mortality through the pathway of decreasing over-production of PGE_2_.

## Data Availability

The datasets generated and/or analyzed during the current study are not publicly available out of protection for individual participants privacy, but de-identified data may be available from the corresponding author on reasonable request.
